# Regenerative artificial intelligence in *Journal of Biological Chemistry*

**DOI:** 10.1016/j.jbc.2023.105008

**Published:** 2023-07-20

**Authors:** Roger J. Colbran, Alex Toker

One cannot read or listen to the news each day without some commentator informing the world that the application of generative artificial intelligence technologies is revolutionizing how we all work and play by creating content such as text, images, audio, and synthetic data. The scientific community is no exception, and barely a day goes by without at least one discussion with a colleague on the potential for ChatGPT and other platforms to positively (or negatively) impact how we approach scientific writing, whether for grant applications, scientific papers, reviews, exams, or other academic purposes. The relative merits and concerns about the use of these tools have been extensively discussed in commentaries and news articles in recent months (1, 2, 3, 4, 5, 6). While on one hand generative AI-based text tools can quickly generate useful summaries of complex topics, there are also abundant anecdotal horror stories about how citations are “invented” to support what may initially seem to be plausible positions on a range of topics. As AI technology continues to evolve rapidly and its uses expand dramatically, the *Journal of Biological Chemistry* is releasing a position statement to define appropriate and inappropriate uses of AI-based chat technologies in the preparation and review of manuscripts being considered for publication.

## Writing

The use of generative AI technologies during manuscript preparation would seem to have strong potential to improve the clarity and quality of manuscripts that are submitted for review. This may be particularly beneficial for authors with first languages other than English, perhaps helping to improve overall equity in science (7). For these reasons, we welcome authors to make use of generative AI technologies in editing the text of their manuscript, in much the same way that authors may make use of more traditional editing services (several commercial editing services are listed in the JBC instructions to authors). In both cases, it is the author’s responsibility to ensure that this process does not alter the intended meaning and that appropriate (and real) citations are used as needed. We now require that the use of such services should be transparently and specifically declared in the acknowledgment section of submitted manuscripts. Importantly, it is inappropriate for ChatGPT or similar technologies, or more traditional commercial editing services, to be included as a co-author, because such technologies/services cannot meet all of the essential authorship requirements.

• Substantial contributions to conception and design, acquisition of data, or analysis and interpretation of data;
• Drafting the article or substantively contributing to revisions in intellectual content;
• Final approval of the version to be published;
• Agreement to be accountable for all aspects of the work.

## Review

An argument can be made that the uses of generative AI text technologies could similarly improve the peer review process, if only by enhancing the quality and clarity of manuscript reviews and decision letters (which authors might greatly appreciate!). However, confidentiality is a core principle of the peer-review process. Numerous concerns regarding the ChatGPT data retention policies have been identified (4) (https://www.makeuseof.com/chatgpt-privacy-issues/), including allowing retained conversations to be accessed by their AI trainers to improve future performance. Thus, uploading any part of the manuscript into a public AI tool may violate the authors’ confidentiality and proprietary and/or data privacy rights, and manuscripts should not be evaluated using generative AI tools. Similarly, these tools should not be used by reviewers, editorial board members, or senior editors to assist in preparing critiques or decision letters. In short, the confidentiality requirements for peer review of manuscripts take precedence over any benefit that may or may not be gained by using generative AI technologies for these purposes.

We have updated the JBC Guide for Authors to explain our principles for the use of generative AI technologies by authors, peer reviewers and editors. These guidelines are explained in more detail on our publisher's ethics page. Note that we anticipate that our guidelines may be further updated to reflect the rapid ongoing evolution of these technologies. Furthermore, we will be carefully watching the developing ability of generative AI to “create” raw image data, potentially including gels, blots, and micrographs, that may be difficult to detect using our current image forensics pipeline, potentially leading to the need to provide additional guidance on the use of these tools.
